# 达芬奇机器人手术系统与电视胸腔镜在胸内纵隔疾病手术治疗中的对比研究

**DOI:** 10.3779/j.issn.1009-3419.2014.07.11

**Published:** 2014-07-20

**Authors:** 仁泉 丁, 向东 童, 世广 许, 大坤 张, 昕 高, 洪 滕, 家骐 曲, 述民 王

**Affiliations:** 110016 沈阳，沈阳军区总医院胸外科 Department of Thoracic Surgery, General Hospital of Shenyang Military Region, Shenyang 110016, China

**Keywords:** 达芬奇机器人手术系统, 纵隔疾病, 微创手术, Da Vinci robot system, Mediastinal lesions, Minimally invasive surgery

## Abstract

**背景与目的:**

近年来达芬奇机器人手术系统（da Vinci robot system）应用于治疗胸内纵隔疾病日趋成熟。本研究通过总结沈阳军区总医院近3年来在纵隔疾病中行手术治疗的临床病例资料，探讨达芬奇机器人手术系统在手术中的疗效；并比较其与电视胸腔镜在纵隔手术中的优缺点，展望达芬奇机器人手术系统在纵隔手术中的应用前景。

**方法:**

选择2010年1月-2013年11月沈阳军区总医院行达芬奇机器人手术及电视胸腔镜下（含胸腔镜辅助小切口）手术的胸内纵隔疾病患者共203例。对两组的手术时间、术中失血量、术后3天内引流总量、术后拔管时间、术后住院时间、手术费用进行比较，结果应用SPSS 19.0进行相关分析。

**结果:**

两组共203例患者均顺利完成手术。术后恢复良好，无围手术期死亡病例。手术时间机器人组为82（20-320）min，电视胸腔镜组89（35-360）min，差异无统计学意义（*P*＞0.05）。术中出血量：机器人组为10（1-100）mL，电视胸腔镜组50（3-1, 500）mL；术后72 h引流量：机器人组215（0-2, 220）mL，电视胸腔镜组350（50-1, 810）mL；术后拔管时间：机器人组3（0-10）d，电视胸腔镜组5（1-18）d；术后住院天数：机器人组7（2-15）d，电视胸腔镜组9（2-50）d；手术费用：机器人组（18, 983.6±4, 461.2）元，电视胸腔镜组（9, 351.9±2, 076.3）元，以上指标两组比较差异均具有统计学意义（*P*＜0.001）。

**结论:**

达芬奇机器人手术与电视胸腔镜手术在胸内纵隔疾病的手术时间相当，在手术安全性以及术后快速恢复上均优于胸腔镜手术，但手术费用也比胸腔镜手术明显增加。

## Abstract (en)

**Background and objective:**

In recent years, Da Vinci robot system applied in the treatment of intrathoracic surgery mediastinal diseases become more mature. The aim of this study is to summarize the clinical data about mediastinal lesions of General Hospital of Shenyang Military Region in the past 4 years, then to analyze the treatment effect and promising applications of da Vinci robot system in the surgical treatment of mediastinal lesions.

**Methods:**

203 cases of mediastinal lesions were collected from General Hospital of Shenyang Military Region between 2010 and 2013. These patients were divided into two groups da Vinci and video-assisted thoracoscopic surgery (VATS) according to the selection of the treatments. The time in surgery, intraoperative blood loss, postoperative drainage amount within three days after surgery, the period of bearing drainage tubes, hospital stays and hospitalization expense were then compared.

**Results:**

All patients were successfully operated, the postoperative recovery is good and there is no perioperative death. The different of the time in surgery between two groups is Robots group 82 (20-320) min and thoracoscopic group 89 (35-360) min (*P* > 0.05). The intraoperative blood loss between two groups is robot group 10 (1-100) mL and thoracoscopic group 50 (3-1, 500) mL. The postoperative drainage amount within three days after surgery between two groups is robot group 215 (0-2, 220) mL and thoracoscopic group 350 (50-1, 810) mL. The period of bearing drainage tubes after surgery between two groups is robot group 3 (0-10) d and thoracoscopic group: 5 (1-18) d. The difference of hospital stays between two groups is robot group 7 (2-15) d and thoracoscopic group 9 (2-50) d. The hospitalization expense between two groups is robot group (18, 983.6±4, 461.2) RMB and thoracoscopic group (9, 351.9±2, 076.3) RMB (All *P* < 0.001).

**Conclusion:**

The da Vinci robot system is safe and efficient in the treatment of mediastinal lesions compared with video-assisted thoracoscopic approach, even though its expense is higher.

目前，常规开胸手术和电视胸腔镜手术（video-assisted thoracoscopic surgery, VATS）是治疗胸内纵隔疾病主要手段。常规开胸手术创口巨大，还需使用撑开器撑开，术后切口疼痛强烈、恢复时间长、切口瘢痕明显以及并发症较多。近些年来随着电视胸腔镜技术的发展，其创伤较小、术后恢复较快、术后出现并发症较少等优势使其在普胸外科广泛应用。目前大部分胸内纵隔病变都能够通过电视胸腔镜手术切除，但是由于电视胸腔镜多为单镜头，视野相对狭窄，对一些位置较深，操作空间较小的病变，器械之间相互干扰严重，手术操作难度大，使手术适应症受到限制。然而，对于微创外科的新宠儿达芬奇机器人手术系统来说，上述问题迎刃而解。

医用机器人的研究最早始于20世纪中叶，达芬奇-S外科手术系统最早于1997年研制成功，2000年通过美国食品药品监督管理局（Food and Drug Administration, FDA）认证后应用于临床外科。该手术系统能向术者提供广角高清实时同步的术野图像，机械手臂的自由活动度完全超越人体关节活动度，并且在稳定性上滤除主刀医生的手部颤抖，动作更为精细，使手术的安全性进一步提高。

本研究回顾性分析2010年1月-2013年11月在沈阳军区总医院胸外科行达芬奇机器人手术及电视胸腔镜（含胸腔镜辅助小切口）手术的胸内纵隔疾病患者共203例，对两组的手术时间、术中失血量、术后3天内引流总量、术后拔管时间、术后住院时间、手术费用进行比较。

## 1 资料与方法

### 1.1 资料

2010年1月-2013年11月我院203例胸内纵隔肿物患者行胸腔镜或机器人手术治疗。

#### 1.1.1 达芬奇机器人组

共120例，男性63例，女性57例，中位年龄48岁，年龄范围17岁-83岁。病理证实为纵隔囊肿54例，胸腺瘤36例，神经源性肿瘤13例，畸胎瘤7例，还包括结节病3例、非典型增生2例、血管瘤2例、淋巴结增生2例、结核1例。

#### 1.1.2 电视胸腔镜组（含胸腔镜辅助小切口）

共83例，男性41例，女性42例，中位年龄50岁，年龄范围13岁-79岁。病理证实为纵隔囊肿34例，胸腺瘤31例，神经源性肿瘤6例，畸胎瘤3例，还包括结节病3例、淋巴结增生3例、结核3例。入选病例在性别、年龄、病理类型等方面具有可比性，两组差异无统计学意义（*P*＞0.05）。二组病例术前影像学均无明显外侵表现，根据自身经济情况自愿选择手术方式。203例全部顺利完成手术，无中转开胸。

### 1.2 方法

#### 1.2.1 机器人组

##### 1.2.1.1 麻醉、体位及切口选择

本组患者全部采用双腔气管插管、全身麻醉、健侧肺通气、健侧卧位，患者双上肢屈曲、抱枕，部分患者需调整手术床使躯干成折刀位，使肋间隙被动增宽。切口选择的原则是开口于肿瘤方向的三角形，两侧器械臂孔距离进镜口与病变连线的距离至少8 cm，避开上肢、肩胛等结构。当病变位于胸膜顶处（图 1）：折刀位，于患侧腋中线第6肋间行1.2 cm切口进镜，腋前线3肋间和肩胛下线与腋后线之间第6肋间行0.8 cm切口入操作臂。病变位于前上纵隔处（图 2）：略后仰，于患侧腋后线第6肋间行1.2 cm切口进镜，腋中线3肋间和腋前线第5或第6肋间行0.8 cm切口入操作臂。病变位于前下纵隔处：略后倾，于患侧腋后线第6肋间行1.2 cm切口进镜，腋前线4肋间和腋前线第7或第8肋间行0.8 cm切口入操作臂。病变位于后上纵隔处：略前倾，于患侧腋前线与锁骨中线之间第5肋间行1.2 cm切口进镜，腋中线第3肋间腋后线与肩胛下线之间第7肋间行0.8 cm切口入操作臂。病变位于后下纵隔处：略前倾，于患侧腋前线与锁骨中线之间第6肋间行1.2 cm切口进镜，腋中线第4肋间和腋中线第8肋间行0.8 cm切口入操作臂^[1]^。

**1:**
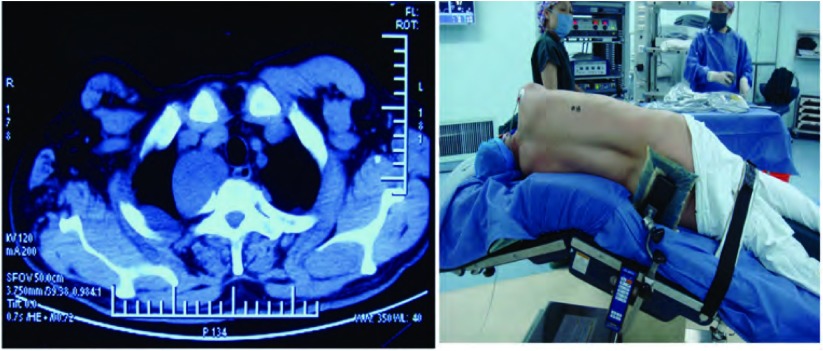
右上纵隔近胸膜顶肿物，采用折刀位。 The right superior mediastinum near apical pleural tumor, the fold.

**2:**
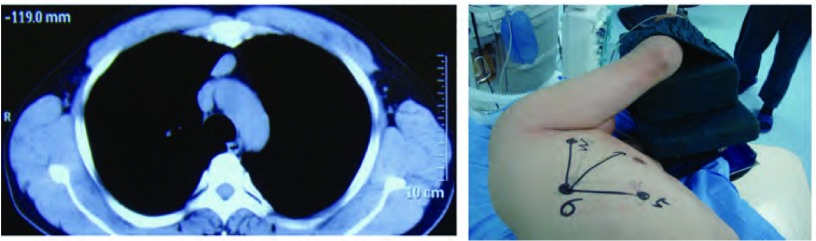
右前上纵隔肿物，采用健侧卧位，略后倾，双手屈曲抱枕。 Right anterior mediastinal mass, the contralateral decubitus, slightly backward, both hands buckling.

##### 1.2.1.2 手术过程

胸外科常规术区消毒、铺一次性无菌单，于标记位置切口置入trocar后送入镜头，探查胸腔内无广泛粘连后建立8 mmHg人工气胸。于内镜引导下分别切口并置入两个操作臂戳卡，从进境口与肿物连线方向推入床旁操作臂系统并戳卡链接。一般右手臂采用单极电凝钩，左手臂采用双极电凝钳，在病变包膜外层水平进行完整游离，若考虑病变为胸腺瘤并且合并重症肌无力则行全胸腺整体切除及前纵隔脂肪清扫，创面确切止血后，将病变取出。取标本通常不另作切口，撤出一个手臂及戳卡后经此切口取出，若病变为较大的实性肿物，则需将操作孔扩大2 cm-3 cm，必要时在标本袋中将病变分解。根据情况选择是否置入胸引管，一般于进境口置管。待肺复张良好后，缝合各操作孔。

#### 1.2.2 电视胸腔镜组

##### 1.2.2.1 麻醉、体位及切口选择

本组患者全部采用双腔气管插管、全身麻醉、健侧肺通气、健侧卧位。切口选择于患侧腋中线第6或第7肋间进胸腔镜，病变位于上、中纵隔则行腋中线第4肋间和腋后线第5肋间各行1.5 cm切口入器械，或腋中线4肋间3 cm-7 cm小切口入胸，病变位于下纵隔则行腋后线至腋中线行3 cm-7 cm小切口入胸。

##### 1.2.2.2 手术过程

胸外科常规术区消毒、铺一次性无菌单，于标记位置切口置入trocar后入视频镜头，观察胸腔内有无广泛、致密粘连，探查病变位置、大小及与周围组织的关系。用电凝钩打开病变边缘胸膜，仔细分离病变，以小纱球钝性分离。如遇较大滋养血管则以电凝或超声刀切断。其余处理同机器人手术。

### 1.3 统计学方法

收集两组的手术时间、术中失血量、术后3天内引流总量、术后拔管时间、术后住院时间、手术费用进行比较。所有数据均采用SPSS 19.0软件进行分析，符合正态分布的计量资料用均数±标准差描述，不符合正态分布的计量数据使用中位数和范围描述，计数资料以百分比表示。组间比较采用独立样本*t*检验，卡方检验或秩和检验。*P*＜0.05为差异具有统计学意义。

## 2 结果

两组共203例患者均顺利完成手术。术后无严重并发症，均恢复良好，无围手术期死亡病例。手术时间：机器人组82（20-320）min，胸腔镜组89（35-360）min，两组差异无统计学意义（*P*＞0.05）。术中出血量：机器人组10（1-100）mL，胸腔镜组50（3-1500）mL，两组差异具有统计学意义（*P*＜0.001）。术后3天内引流量：机器人组215（0-2, 220）mL，胸腔镜组350（50-1, 810）mL，两组差异具有统计学意义（*P*＜0.001）。术后拔管时间：机器人3（0-10）d，胸腔镜组5（1-18）d，两组差异具有统计学意义（*P*＜0.001）。术后住院天数：机器人组7（2-15）d，胸腔镜组9（2-50）d，两组差异具有统计学意义（*P*＜0.001）。手术费用：机器人组为（18, 983.6±4, 461.2）元，胸腔镜组为（9, 351.9±2, 076.3）元，两组差异具有统计学意义（*P*＜0.001）。

达芬奇机器人手术系统与电视胸腔镜在胸内纵隔疾病手术的比较中，手术时间的比较无统计学差异（*P*＞0.05），在术中失血量、术后3天内引流量、术后拔管时间、术后住院时间、手术费用的比较中具有统计学差异（*P*＜0.05），见表 1。其中，机器人手术在术中失血量、术后3天内引流总量、术后拔管时间、术后住院时间的指标中均少于胸腔镜手术，说明机器人手术在手术的安全性以及术后快速恢复上均优于胸腔镜手术，但手术费用比胸腔镜手术明显增加。

**1:** 两组患者临床资料 The two groups of patients with clinical data

Index	Robot group (*n*=120)	Thoracoscopic group (*n*=83)	Test statistics	*P*
Operation time (min)	82 (20-320)	89 (35-360)	Z=-0.676	＞0.05
The amount of bleeding (mL)	10 (1-100)	50 (3-1, 500)	Z=-7.383	＜0.001
Drainage in 3 days after operation (mL）	215 (0-2, 220)	350 (50-1, 810)	Z=-5.133	＜0.001
Time to extubation after surgery (d)	3 (0-10)	5 (1-18)	Z=-4.713	＜0.001
Postoperative hospital stay (d）	7 (2-15)	9 (2-50)	Z=-5.754	＜0.001
Operation cost（yuan）	18, 983.6±4, 461.2	9, 351.9±2, 076.3	*t*=20.6	＜0.001

## 3 讨论

达芬奇机器人外科手术系统是当代最先进的外科手术系统，是由Intuitive Surgical公司研制开发的，目前正在以飞跃式发展。此手术系统结合多种新兴前沿学科知识与工艺，从而使外科手术的微创化、高智能化和高数字化得以成为现实。机器人最初的设计是为实现在心胸外科手术中的微创化，逐渐普及到泌尿外科、肝胆外科、妇产科、胃肠外科、耳鼻喉等学科^[2]^。据Chindex Medical有限公司统计，截止2014年3月，全球的达芬奇系统装机量是3, 039台，其中美国2, 116台，亚洲311台。近年来达芬奇机器人手术以每年近10万例的速度呈现跳跃式增长。

胸内纵隔疾病手术术式较多，现在仍然没有一种固定的术式成为标准术式。普遍认为常规开胸手术视野广，操作空间开阔，手术能够完整的切除病变。但这种术式切口长，创面大，渗出多，对于一些长期应用糖皮质激素的患者，同时合并骨质疏松，可能进一步影响切口恢复，如合并重症肌无力，术后可能出现重症肌无力危象，导致呼吸肌麻痹、呼吸骤停的可能^[3]^。如行劈胸骨术式因切口较长，影响美观，一些年轻女性患者，不能接受这种开胸手术，若将切口下移，不完全切开胸骨，在这种情形下撑开胸骨受限，术区暴露不充分，使胸腺上极的处理受到限制，手术效果不能肯定。

近20年来，随着人们对微创手术的追求，电视胸腔镜手术逐渐被接受、认可，越来越多的患者及手术医师选择胸腔镜手术切除纵隔内病变。因其治疗效果与常规开胸相当，且在手术后平均住院时间、术中失血量以及患者对术式的接受程度均优于常规开胸术式。故多家胸外科已将电视胸腔镜手术作为治疗胸内纵隔疾病的首选方法^[4]^。但电视胸腔镜手术还存在一些固有缺点，胸腔镜为二维成像，没有立体感，整体视觉感受差，容易误操作，在处理上纵隔及胸膜顶病变时空间狭小，操作困难，器械相互干扰严重。再者，胸腔镜器械的力臂较长，手术医师在长时间手术中可能出现抖动，在这种情况下由于杠杆原理动作被放大，增大了对周围组织副损伤的几率。尤其在纵隔这个心脏、大血管及神经聚集的区域，这种副损伤可能产生严重的后果。对于胸腔镜手术切除纵隔内病变的问题上，各家仍存争议，有学者^[5, 6]^认为对于一些病理回报为低度恶性的纵隔肿瘤，如果切除范围不够大，无瘤效果就不确切，可能存在肿瘤种植转移，还有学者^[7, 8]^认为电视胸腔镜的切除例数较少，手术经验较少，没有大量常规开胸手术经验的手术医师不应做腔镜手术。

达芬奇机器人外科手术系统在手术控制，手术操作的稳定性、灵活性、精细度，以及对术野观察度上弥补了电视胸腔镜固有的不足。达芬奇机器人外科手术系统同时具有对术野的3D立体成像^[9]^、术野组织放大10倍-15倍，操作臂腕部有7个自由度，能够模拟手和手腕的动作，比人手的5个自由度更加灵活^[9-11]^，并且能够通过多种器械，完成不同的操作任务，如转动、缝合、夹闭等操作。同时振动消除系统和动作定标系统，可保证机械臂在狭小的术野内进行精确的操作^[12, 13]^。

近4年来，北美及西欧30多个医院采用达芬奇机器人行纵隔病变手术6, 000余例^[14]^。本研究通过对术中出血量、术后3天内引流量、术后拔管时间、术后住院天数的临床资料比较看出机器人组在胸内纵隔病变手术的安全性及术后恢复速度上要明显优于胸腔镜组。在手术时间上，两组差异不大，但电视胸腔镜作为一项已经成熟的手术技术，在手术时间上基本难以提高。而机器人手术是一项新兴的手术技术，随着病例的不断积累，手术技巧的不断提高，在缩短手术时间上提高的空间还很大。在手术费用上机器人组明显高于胸腔镜组，主要是需要使用专有的手术器械，由于是专利技术，生产机器人器械的厂家现阶段处于市场垄断地位，缺少竞争导致价格居高不下。这也使经济条件一般的患者，望机器人兴叹。但随着人们收入的增长，医疗保险普及范围的增大以及报销比例的增加，手术费用已经能够被大多数患者所接受。再者，国内已开始自主研发医用手术机器人系统，相信在不久就会对现有的机器人手术系统的市场价格形成挑战。个人认为本系统最大缺陷是缺乏力反馈系统^[15]^，在处理血管过程中易因过度牵拉撕裂血管壁而造成出血^[16]^。随着手术经验的积累和手术熟练程度的提高，这种情况在很大程度上是可以避免的^[17]^。随着系统的不断更新和技术的进一步完善，达芬奇机器人外科手术系统以其创伤小、术中出血少、术后恢复快等优势越发的受到青睐。机器人手术在胸内纵隔疾病的外科治疗方面将会得到更广泛的应用，发挥更积极的作用^[18]^。
